# Objective Detection of Retinal Vessel Pulsation

**DOI:** 10.1371/journal.pone.0116475

**Published:** 2015-02-02

**Authors:** William H. Morgan, Anmar Abdul-Rahman, Dao-Yi Yu, Martin L. Hazelton, Brigid Betz-Stablein, Christopher R. P. Lind

**Affiliations:** 1 Lions Eye Institute, University of Western Australia, Nedlands, Australia; 2 Statistics and Bioinformatics Group, Institute of Fundamental Sciences, Massey University, Palmerston North, New Zealand; 3 Neurofinity, School of Surgery, University of Western Australia, Nedlands, Australia; 4 Department of Ophthalmology, Manukau Health, Auckland, New Zealand; University of Melbourne, AUSTRALIA

## Abstract

**Purpose:**

Retinal venous pulsation detection is a subjective sign, which varies in elevated intracranial pressure, venous obstruction and glaucoma. To date no method can objectively measure and identify pulsating regions.

**Method:**

Using high resolution video-recordings of the optic disk and retina we measured fluctuating light absorption by haemoglobin during pulsation. Pulsation amplitude was calculated from all regions of the retinal image video-frames in a raster pattern. Segmented retinal images were formed by objectively selecting regions with amplitudes above a range of threshold values. These were compared to two observers manually drawing an outline of the pulsating areas while viewing video-clips in order to generate receiver operator characteristics.

**Results:**

216,515 image segments were analysed from 26 eyes in 18 research participants. Using data from each eye, the median area under the receiver operator curve (AU-ROC) was 0.95. With all data analysed together the AU-ROC was 0.89. We defined the ideal threshold amplitude for detection of any pulsating segment being that with maximal sensitivity and specificity. This was 5 units (95% confidence interval 4.3 to 6.0) compared to 12 units before any regions were missed. A multivariate model demonstrated that ideal threshold amplitude increased with increased variation in video-sequence illumination (p = 0.0119), but between the two observers (p = 0.0919) or other variables.

**Conclusion:**

This technique demonstrates accurate identification of retinal vessel pulsating regions with no areas identified manually being missed with the objective technique. The amplitude values are derived objectively and may be a significant advance upon subjective ophthalmodynamometric threshold techniques.

## Introduction

Retinal venous pulsation is an important and long recognised clinical sign,[1] which becomes absent in subjects with elevated intracranial pressure,[2] [3] retinal venous occlusion[4, 5] and in many glaucoma patients.[6–8] Currently, its presence is subjectively determined by an observer, who may induce pulsation, if absent, by elevating the intraocular pressure. This was first described in 1853[1] and the threshold intraocular pressure required to induce venous pulsation has been found to be strongly predictive of intracranial pressure[3], glaucoma progression[9] and severity of venous occlusion.[5] Unfortunately, the utility of venous pulsation assessment suffers from subjectivity, variability and the need for a skilled observer.[10]

There is a need for an observer-independent technique capable of objectively measuring an aspect of vessel pulsation across the optic disc and surrounding retina. Such a technique needs to be verified against expert clinical observation. Receiver operating characteristics have been shown to be robust in assessing accuracy of image analysis techniques in the detection of clinical features.[11, 12]

Current recording techniques for assessing retinal vessel pulsation use either direct video recordings with an observer viewing the recordings and commenting upon the presence or absence of pulsation,[13, 14] or use an image analysis technique detecting vessel diameters.[15] The latter technique requires an observer to outline the vessels of interest and diameter measurements are calculated from the video frames. It will measure vessel diameters on the retinal surface but not on the optic disc.[15] We have recently described a modified photo-plethysmographic technique for measuring retinal vessel segment pulsatility, which also measures pulsatility from optic disc surface vessels.[16] The reproducibility measurements with this technique demonstrate a coefficient of variation 13% for vessel pulsation amplitude and 4% for pulsation timing.

We modified this system to objectively measure vessel pulsation amplitude from sectors of retinal images. This was done using short retinal video recordings, which can also be viewed by experienced observers to manually ascertain vessel pulsation. We present results of our objective technique and its comparison with two observers.

## Methods

This study was performed under the aegis and approval of the University of Western Australia Human Ethics Committee adhering to the tenets of the Declaration of Helsinki. Written consent was obtained from each of the participants. Healthy adult research participants were recruited from the medical student body and relatives of patients treated at the Lions Eye Institute.

The modified plethysmographic technique and principles have been described previously.[16] We briefly describe the technique along with the modifications. A pulse oximeter is connected to the patient so that the cardiac cycle timing was recorded on the audio trace of the video segments in a manner described previously.[17] This enables mathematical analysis of the periodic component in time with the cardiac cycle.

Participants were required to have clear ocular media, with no retinal or optic nerve pathology and normal visual fields as assessed by Humphrey standard automated perimetry or Frequency Doubling Perimetry (Humphrey—Zeiss, Dublin, Ca). Subjects were not excluded on the basis of smoking history or systemic hypertension. They all had visual field testing for one half hour prior, then dilated for another half hour. They rested and were not given caffeine during this period. Blood pressure was not measured. Written consent was obtained from each of the participants. The participant’s pupils were dilated with 1% Tropicamide. Following dilation a pulse oximeter (Nellcor N65, Covidien, Mansfield, MA) was applied to the right index finger. Each participant underwent video recording with a non-contact 60 dioptre indirect lens in the first instance. When there was excessive eye movement a Meditron ophthalmodynamometer (Meditron, Voeklingen, Germany) was used because it stabilises eye movements. The ophthalmodynamometer was used with a Goldmann three-mirror contact lens. Contact gel was applied to the lens prior to contact with the cornea. The participant sat at a video slit-lamp (Carl Zeiss, West Germany) and had video recordings taken (Canon 5D Mark III, Japan). This camera has low noise at low light levels and allows video-recordings to be taken at lower light levels than our previous system.[18] This allowed some subjects to maintain fixation during video recording with the 60dioptre lens. Several sequences of at least three cardiac cycles in length were taken. In several subjects recordings were taken from both eyes. The camera has an inbuilt microphone and the pulse oximeter was placed 10cm from the camera so that it recorded the pulse “beat” concurrently with the video recordings. The pulse oximeter audio transmission time to camera is estimated at 0.3ms and the camera analog to digital conversion estimated to take 1ms leading to an approximate 1.3ms delay. The pulse oximeter was placed in an identical position for all recordings so that this time delay was constant and the audio pulse recorded used to mark the start point of each cardiac cycle.

**Figure 1:**
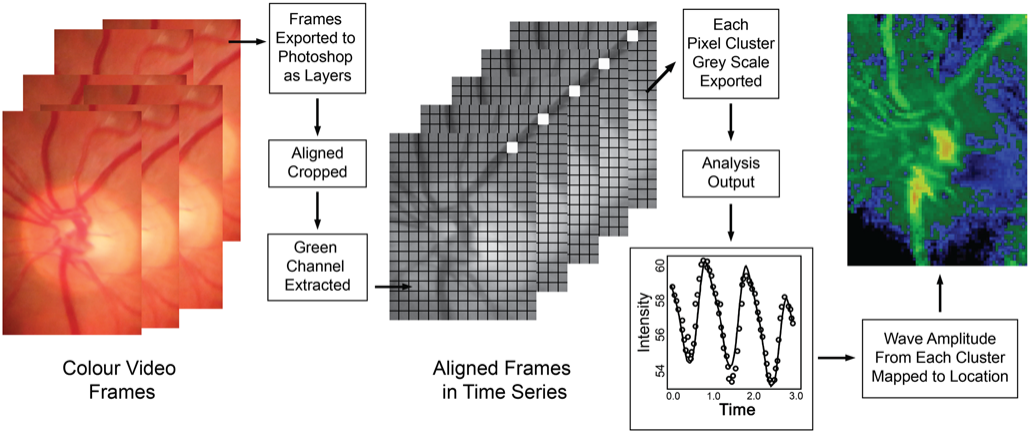
Schematic diagram of the technical sequence for objectively analysing the video frames, calculating pulsation amplitudes and generating heat maps of amplitude.

High-quality single videos, with frame rate of 25 fps, each lasting three cardiac cycles was extracted from each raw recording session and used for automated and manual analysis. For automated analysis, the video frames were exported as separate images in .tif format. These were imported as layers (separate frames) into Photoshop (CS6, San Jose, CA). The portion of the images containing the optic disc and surrounding retina was selected and the surround cropped (Fig. 1). An automatic image alignment algorithm was applied to align the images in order to centre the optic disc and blood vessels. This algorithm uses a composite affine transformation, combining shear, linear and rotational shifts to match a reference layer to the other layers. This layered image set was kept aside and the degree of movement horizontally and vertically between the two most displaced image layers was used to calculate the degree of image wobble. The aligned image series was then cropped again simply to remove extraneous portions of image that were not consistently visible in all layers.

These cropped aligned images from three cardiac cycles were imported into custom written software (written in R language)[19] where the green colour channel from each image layer was extracted. The optical path length through haemoglobin and its relationship to the transmission of light is described by the Beer-Lambert law, which demonstrates that the path length is proportional to the negative logarithm of transmittance. Each image was broken into an array of 5x5 pixel green channel intensity values. Each intensity value was transformed by taking its negative logarithm so the resulting parameter would have a stronger linear relationship to the optical path length. This transformation of the values also rendered variations in camera gamut linear rather than exponential. We calculated an approximation for optical path length absorbance by hemoglobin in microns assuming that intensity recorded was associated with transmittance, an extinction co-efficient of 12 L mmol^-1^ cm^-1^ at 550 nanometres for both oxy and de-oxy haemoglobin[16, 20] and an average hemoglobin concentration of 150g/L in our participants along with a molar density of 64,500 grams haemoglobin per mole. We divided this result by 2 assuming that the bulk of light passes through and is reflected back through the same vessel encountered meaning that the optic path length is twice the actual vessel depth. This resulted in a factor of 68, which was then multiplied with the negative logarithm. The mean of that 5x5 pixel cluster of transformed intensity values was stored. Our results are presented in arbitrary units and we do not claim that they necessarily reflect accurate change in vessel depth in microns.

**Figure 2:**
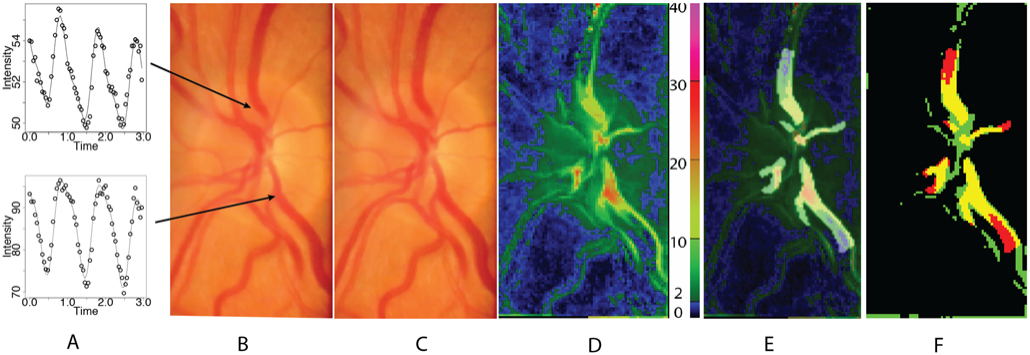
A diagram showing 2 cluster waveforms from different optic disk regions over three cardiac cycles (A) with video frames taken during diastole (B) and systole (C). A heat map with colour scale (D) is shown with observer 1 manual outline of pulsating region overlaid upon the heat map (E). Objective detection (F) with threshold amplitude set at 5 units is shown in yellow where this was in agreement with observer, in green without agreement from observer and in red where the observer noted pulsation but amplitude values were less than 5 units.

The software then created a three-dimensional matrix comprising a series of two-dimensional images as a stack containing the green channel information (Fig. 1). Each layer in the stack contained information which mapped directly to corresponding video frame and so spatial coherence was maintained. The stack contained the time information and the aligned clusters were analysed using a harmonic regression assuming cardiac cycle periodicity. A linear spline term was incorporated in the regression to account for the effects of patient movement during video capture. The fitted curves were differentiated and the maximum and minimum values extracted with amplitude calculated for each cluster region. An example of the curves, image and heat map of amplitudes are seen in Figs. 1 and 2. The amplitude values were stored in a separate array to be used for future comparison. Those arrays were used to generate heat maps and one is shown in Fig. 2c with colour coded pixels representing the degree of amplitude measured at each cluster point on the image. Two original aligned image frames, one from diastole and the other during systole are shown also in Fig. 2 for comparison and one can see how the amplitude maps have peak values in the location of larger and more central veins.

Two observers viewed the raw three cardiac cycle video sequences. Both observers are experienced ophthalmologists with Observer 1 having a large research interest and expertise in retinal venous pulsation with Observer 2 being newly introduced to this field. The observers were given a cropped frame on a computer screen, taken from the series analysed with which to outline areas of visible pulsation using a cursor tool in Photoshop (Adobe Photoshop CS6, San Jose, CA). On an adjacent screen they viewed the original video clip of the three cardiac cycles and were allowed to view this as often as they wished. The observers outlined corresponding sections on the selected video frame where pulsation was occurring. The section of image contained within their outline was considered to be pulsating by the observers and regions outside of selected areas were deemed to be non-pulsatile by the observers. The image outlined by the observers was scaled down by 5:1 in order to match the automated heat map and amplitude arrays. Any 5x5 pixel cluster through which an observer had considered at least one pixel to be pulsating was considered pulsatile and hence deemed a pulsatile region. These images derived from the observers were converted to a binary array of values either positive (1) for detection of pulsation or negative (0) for the absence of pulsation. Cohen’s kappa statistic was used to calculate the degree of agreement between the two observers.

The observers’ pulsation array and the objective photo-plethysmographic amplitude array were then compared. An amplitude scale of 0:40 was selected for the photo-plethysmographic data because this contained 99.98% of all amplitude values across the images. The objective system created a series of arrays at each integer amplitude threshold value between 0 and 40. Amplitude values greater than threshold were assumed to represent pulsating regions and amplitude values of less than or equal to threshold were assumed to represent non-pulsatile regions. If the cluster region amplitude was above threshold and the observer graded this region pulsation positive then it was scored as true positive (TP) but if the observer graded it as pulsation negative then it was graded as false positive (FP). If the cluster amplitude was below threshold and the observer graded that region as pulsation positive it was considered false negative (FN) but if the observer graded that region as pulsation negative it was graded as true negative (TN).

We calculated the true positive rate (TPR) equal to TP ÷(TP + FN) and false positive rate (FPR) was equal to FP ÷ (FP + TN). Sensitivity is equal to TPR and specificity is equal to 1-FPR. The area under the receiver operating curve (AU-ROC) was calculated empirically for each observer using data from each threshold setting from each subject. Also the data from all subjects was collated and the same calculation of AU-ROC made.

The **ideal threshold** was calculated by selecting the threshold at which specificity and sensitivity were maximal as determined by the maximum product of sensitivity and specificity at any particular threshold. This was calculated in each subject for each observer. The numbers of pulsating regions identified by each observer were calculated. For each region the maximum amplitude value contained within a region was noted. This was the value above which pulsation would be missed if the selected threshold amplitude had been chosen above this level. This value was termed the **critical amplitude**.

For each series the average green channel intensity for the entire frame was calculated. The mean and standard deviation of intensity over the three cardiac cycles was calculated for each video clip. The standard deviation was used as an index of how varied the illumination had been during each video recording.

For each series of images, when the initial individual frames were aligned, the maximum vertical and horizontal displacement of the frames in pixels was noted prior to cropping the series. Using Pythagoras’ formula, displacement was calculated as square root of vertical displacement squared plus horizontal displacement squared, yielding an index of image movement during the recording.

### Statistical Analysis

All data is presented as a mean and standard deviation unless otherwise stated. All analysis was performed using R.[19] The comparison of observers’ identification of pulsation regions was calculated using Cohen’s kappa statistic. Note that all the pixel cluster data from all images was used. All data was tested for normality using the Shapiro-Wilk test, transformed when non-normal then retested. Area under the receiver operator characteristic curve were transformed using a log(1 − AU-ROC) transform. Ideal threshold was transformed using a logarithmic transform. Linear mixed models were used to model the effect of explanatory variables upon either ROC AUC, ideal thresholds and critical amplitude.[21] The explanatory variables used were observer, image illumination variation (standard deviation), image movement, sex, age and use of ophthalmodynamometry. Given that some patients had video recordings from both eyes and that all patients’ video recordings were viewed by the two observers, a random factor for patient identity was used and a separate random factor for right and left eye was nested within the former to account for correlations between right and left eye and correlations between identical patients.

## Results

Twenty-six video recordings from 18 participants (10 male, 8 female) with mean age 52 years (sd 21) were taken. There were 13 right eyes and 13 left eyes with 16 recordings utilizing an ophthalmodynamometer and 10 without. The subjects mean IOP was 16.5 (sd 2.8) mmHg. The ophthalmodynamometric force applied averaged 24 (sd 26) grams, creating a mean increase in IOP of 23mmHg using a previously published calibration constant.[22] The observers determined that all visible pulsation occurred within retinal veins except in one subject. This subject (subject 12) had ophthalmodynamometry applied and this induced arterial pulsation without visible venous pulsation.

The average raw image size was 405 pixels (sd 100) by 502 pixels (sd 174). A total of 216,529 5x5 pixel clusters were analysed. The two observers viewed a mean 2.4 pulsating regions from each subject’s video sequence (sd 1.1) with Observer 1 seeing more (3.1) than Observer 2 (1.8). Cohen’s kappa for the detection of pulsating regions between observers was 0.98. The AU-ROC was averaged between the two observers for each individual and this data along with the average ideal threshold and other core data is presented in Table 1.

**Table 1:** Average values of key parameters for each eye: AU-ROC = area under the receiver operator characteristic curve, Ideal Thresh = ideal threshold, Illum sd = image illumination variation and Tot Movt = maximum frame movement during video of three cardiac cycles.

**Eye**	**Age**	**Side**	**AU-ROC**	**Ideal Thresh**	**Critical Amplitude**	**Illum sd**	**Tot Movt**
1	30	r	0.954	4.5	18.0	1.4	63.3
2	30	l	0.986	7	16.0	1.3	73.5
3	26	r	0.853	8	13.9	2.1	40.3
4	26	l	0.894	6	12.0	2.2	74.3
5	23	r	0.949	4.5	13.1	0.8	41.4
6	23	l	0.966	4	12.0	0.7	32.7
7	29	l	0.964	3	12.4	0.5	32.0
8	29	r	0.938	5	15.8	1.0	44.7
9	48	l	0.898	4	10.7	0.9	22.4
10	81	l	0.918	2	7.1	2.0	50.0
11	25	r	0.922	5.5	19.8	0.8	51.7
12	73	r	0.964	2.5	11.6	1.4	39.7
13	78	l	0.979	5	22.7	1.4	26.9
14	40	l	0.984	3.5	9.4	0.6	30.2
15	67	r	0.952	10	26.8	2.5	41.0
16	69	r	0.978	4	9.1	1.7	82.8
17	65	r	0.939	6	15.0	2.1	115.3
18	65	l	0.960	5	23.6	2.9	181.2
19	59	r	0.950	6	11.0	1.5	82.8
20	59	l	0.940	8.5	25.0	3.0	45.2
21	25	l	0.916	6.5	16.4	2.0	66.2
22	70	l	0.883	9.5	16.3	3.5	71.9
23	65	r	0.976	4	15.3	0.8	26.4
24	72	r	0.987	8.5	22.3	3.4	117.6
25	72	l	0.962	6	13.5	1.5	77.0
26	48	r	0.909	4	16.7	1.0	55.6

The AU-ROC data was skewed to the left so a log (1 − AU-ROC) transform was applied which normalised the data. This data was then used to calculate the confidence intervals and also the linear mixed modelling as described above. Choice of observer was not associated with AU-ROC (p = 0.0716). None of the other explanatory variables (image movement, use of ophthalmodynamometry, image illumination variation, sex or age) was found to influence the AU-ROC with the minimum probability value being 0.1175. The mean of the average AU-ROC between the 2 observers was 0.936 with median 0.953 and 95% confidence interval 0.939 to 0.965 for the median.

**Figure 3:**
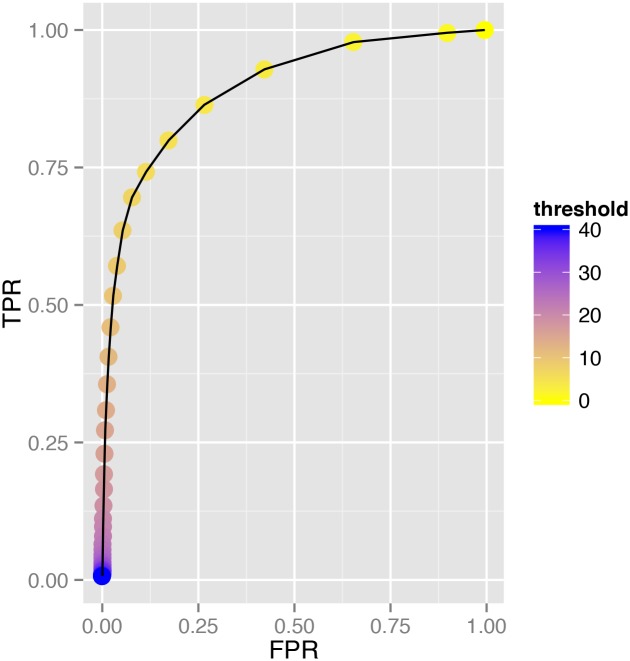
Receiver operator curve for Observer 2 with all data from all eyes analysed together.

When all of the data was combined to create one large array containing all amplitude and threshold values from the 26 image series and all of the observers’ values, the AU-ROC for Observer 1 was found to be 0.889 and an ideal threshold of 5.00. That for Observer 2 was 0.888, again with an ideal threshold of 5.00 (Fig. 3).

The ideal thresholds were skewed to the right so a logarithmic transform was used which normalised this data. The linear mixed modelling with random factors demonstrated that the explanatory variable image standard deviation (illumination variation) was associated with variation in ideal threshold (p = 0.0119) with increased image illumination variation associated with an increased threshold. The choice of observer (p = 0.0919) and age (p = 0.0503) were not significantly associated with ideal threshold. No other explanatory variable (image movement, ophthalmodynamometry use or sex) was significantly associated with ideal threshold either (minimum p = 0.2953). The median ideal threshold was 5 units and the 95% confidence interval calculated using the transformed data was 4.3 to 6.0 units.

The critical amplitude or, amplitude above which, a sector of pulsation would be missed in any participant was found to be higher from Observer 2 (18.9, sd 6.9) compared to Observer 1 (12.6, sd 5.4), p = 0.0002. The data was skewed to the right so a logarithmic transform was used which normalised it. The linear mixed modelling identified that choice of observer was significantly associated with critical amplitude (p = 0.0002) and also variation in illumination (p = 0.0417) was associated but none of the other explanatory variables were associated with variation in critical amplitude.

## Conclusion

This modified photo-plethysmographic technique produces amplitude heat maps, which accurately determine the position of vessel pulsation as confirmed by two independent observers. One observer was a very experienced observer of vein pulsation and saw more pulsating regions than the other senior ophthalmologist who did not have the same vessel pulsation research experience. Pulsating regions identified by the more experienced observer required lower amplitude to be missed most likely because more subtle regions of pulsation were detected. This difference between observers is also reflected in the significant difference between critical pulsation amplitudes required for the objective technique to miss a region of pulsation.

Even though there was this difference between the observers there was no significant difference between receiver operating characteristic area under the curve values in this data set with a median value of 0.953. Additionally, the ideal thresholds were not influenced by identity of observer (p = 0.0919) but were elevated somewhat by variation in video recording illumination (p = 0.0119). The ideal objective threshold was 5 units (95% confidence interval 4.3 to 6.0 units).

When each observer’s findings from 26 eyes were compiled together the AU-ROC was also almost identical (0.88 versus 0.89). They were less than the median of the individual AU-ROC values probably because of variations in image illumination and other factors between the 26 recordings.

The high AU-ROC indicates a high accuracy in detecting the location of pulsating regions. Further, the objective technique is robust because the ideal threshold was significantly lower than any of the critical thresholds. Using the ideal threshold of 5 units resulted in no pulsating regions identified by either observer being missed by the objective technique.

These results demonstrate that this technique can accurately identify the presence and location of retinal vessel pulsation from cardiac cycle timed video recordings. The high AU-ROC suggests that the pulsation amplitude calculated is indicating a useful measure of vessel pulsatility. This objective technique may be useful in settings where detection of retinal vein pulsation has clinical utility, such as in the exclusion of elevated intracranial pressure[23], providing additional information concerning retinal vein occlusion[5] and prognostication in glaucoma.[9] One limitation of this work is that we studied normal volunteers, so any differences in patients with ophthalmic or neurological disorders remains to be seen. The pulse transit time between the heart and finger, where the pulse oximeter is located, can vary by a mean 38ms over 60sec due to blood pressure and other changes.[24] This is equivalent to the timing error of one frame in a video sequence. This appears acceptable and in fact the error is likely to be less than this given that we record for approximately 3 seconds. This variation may reduce the accuracy of the calculated amplitude. Variation in illumination intensity probably also alters the accuracy of calculated amplitude. These results relate principally to the detection of retinal venous pulsation. The arterial pulsation detected in one subject is encouraging but does not demonstrate that this technique can reliably detect arterial pulsation.

The amplitude values themselves are likely to be more useful than a global singular threshold measure gained by ophthalmodynamometry. They may also change during the course of disease. The amplitude heat maps do give an accurate spatial impression of location and degree of pulsation. Individual amplitude values as well as heat maps may be useful in monitoring progress of the above-mentioned disorders.
